# Ischemic preconditioning combined with inter-set palm cooling enhances performance in strength-trained men during high-intensity resistance training: A randomized crossover trial

**DOI:** 10.5114/biolsport.2026.152344

**Published:** 2025-08-13

**Authors:** Chih-Min Wu, Wei-Cheng Chou, Wen-Yi Wang, Zong-Yan Cai

**Affiliations:** 1 Department of Leisure and Sports Management, Cheng Shiu University, Kaohsiung, Taiwan; 2 Graduate Institute of Sports Pedagogy, University of Taipei, Taipei, Taiwan; 3 Center for Physical and Health Education, Si Wan College, National Sun Yat-sen University, Kaohsiung, Taiwan

**Keywords:** Number of repetitions, Total work volume, Peripheral cooling, Lactate, Norepinephrine

## Abstract

This study investigated the effects of ischemic preconditioning (IPC) on performance during highintensity resistance exercise (RE) sessions, as well as the additional effects of inter-set palm cooling (PC) and the potential underlying mechanisms. Twelve resistance-trained men participated in three RE sessions in a randomized order: (1) IPC followed by RE with inter-set PC (15°C for 2.5 minutes), (2) IPC followed by RE, (3) SHAM followed by RE. IPC involved four cycles of 5-minute ischemia/5-minute reperfusion at 220 mmHg on the upper arm, whereas SHAM involved 20 mmHg with a pneumatic cuff. The RE protocol included three sets to exhaustion, consisting of six exercises at 85% of one-repetition maximum. Multiple perceptual parameters were assessed during RE, and blood samples were obtained both before and after four cycles of ischemia/reperfusion as well as after RE. The results indicated that compared with SHAM, IPC significantly increased the total work volume (∆ = 2.6%); the total number of repetitions (∆ = 4.3%); the number of repetitions in specific exercises; and the levels of several arousal indicators, such as norepinephrine levels before RE and arousal level (assessed using the Felt Arousal Scale) during RE (p < 0.05). The IPC + PC combination further outperformed SHAM alone in most parameters (p < 0.05). However, fatigue indicators (rating of perceived exertion and lactate level) did not differ significantly across the protocols. In conclusion, this study suggest that IPC enhances high-intensity RE performance, potentially through increased arousal levels, with PC augmenting exercise performance by amplifying the arousal response.

## INTRODUCTION

Ischemic preconditioning (IPC) is the process of applying a cuff to the upper arm or thigh and then inflating and deflating it in brief cycles to intermittently block and restore blood flow [1–3]. Studies have shown that IPC can boost oxygen delivery by dilating blood vessels and enhance metabolic regulation by activating ATP-sensitive potassium channels, thereby reducing the depletion of energy substrates (ATP and glycogen) and lowering blood lactate (BLa) accumulation [1, 2]. In the context of exercise, IPC was initially implemented in aerobic and anaerobic glycolytic exercises, yielding favorable outcomes [3, 4].

Recent studies on IPC and exercise have increasingly focused on exploring the effect of IPC on both strength and resistance exercise performance. Given the brief ischemia/reperfusion cycles that correspond with IPC principles during muscle contraction [5], along with the involvement of similar metabolic pathways, IPC has been hypothesized to exert beneficial effects on muscle force production [4, 5]. Libonati et al. [5] reported a 14% increase in overall force output during 15 maximal voluntary contractions of the wrist flexors following intermittent ischemia/reperfusion treatments. Similarly, Paradis-Deschênes et al. [6] observed immediate improvements in muscle perfusion and oxygen uptake, resulting in a 12.6% increase in repeated maximal voluntary knee extensions after three cycles of IPC on the lower limb. Carvalho and Barroso [7] discovered a 20% increase in the number of repetitions at 85% of onerepetition maximum (1RM) to exhaustion during a single set of knee extension exercises after four cycles of IPC to the thigh, without alterations in fatigue markers such as the rating of perceived exertion (RPE) and BLa compared to SHAM. Although the exact mechanisms underlying these improvements remain unclear, potential factors include increased reactive hyperemic blood flow [5], muscle perfusion and oxygen extraction capacity [6], and buffering capacity, which may attenuate exercise-induced acidosis [7]. Additionally, da Silva Novaes et al. [8] studied the ergogenic effects of IPC during a resistance training session at 80% of 1RM until exhaustion. This session included six exercises targeting the upper and lower body muscles to simulate real training scenarios. Individuals seeking to increase muscle strength often train at intensities exceeding 85% of their 1RM. The capacity to perform more repetitions at intensities exceeding this threshold—typically quantified as training volume per set— is critical in promoting long-term muscle strength adaptations [9]. While IPC may improve performance in single-joint, high-intensity resistance exercises excereding 85% of 1RM; however, the effects of IPC on high-intensity resistance workouts at 85% of 1RM, including multiple sets and joints, require additional investigation.

In addition to pre-exercise performance enhancement strategies, peripheral cooling has been used as an ergogenic aid during interset rest intervals in resistance exercise, with a focus on a single exercise [9–13]. Peripheral cooling targets specific joints [14, 15] or limb extremities [9–13] to prevent excessive muscle temperature lowering, which can impair nerve and muscle contraction capabilities [16]. Peripheral cooling is commonly applied to the palms [10–12] and the soles of the feet [9, 13] in resistance training investigations because of their greater sensitivity to cold stimuli owing to a dense network of arteriovenous anastomoses [17, 18]. Studies have demonstrated that inter-set palm cooling (PC) can increase the number of repetitions in high-intensity bench press (BP) exercises [10], counteract power output decline during submaximal leg press (LP) flywheel ergometer exercises [11], and increase the number of repetitions in pull-up exercises [12]. Similarly, cooling the feet during rest intervals has been reported to enhance the number of repetitions to exhaustion in high-intensity LP exercises [9] and the maximal LP strength [13]. One possible explanation for how peripheral cooling improves acute resistance exercise performance is that it delays the onset of fatigue by delaying power output decline [11], impeding BLa production [11], and potentially lowering the RPE [10]. Another explanation could be the heightened arousal of the central nervous system causing an increased recruitment of motor units and force production [9, 10, 13, 15]. Norepinephrine (NE), an arousal indicator [19], has not yet been explored in the context of resistance exercise combined with peripheral cooling.

Engaging in resistance training sessions involving multiple sets and exercises over a prolonged duration can cause the development of considerable fatigue. Given the distinct timing and potential mechanisms of IPC (before exercise) and peripheral cooling (during intraexercise rest intervals), it is suggested that combining IPC before exercise with peripheral cooling during exercise could produce a synergistic effect, improving performance during repeated bouts of high-intensity effort. However, the effectiveness of this combination strategy for improving performance during high-intensity resistance exercise involving multiple sets and exercises is currently unclear. Research on peripheral cooling has demonstrated that inter-set PC improves performance in lower and upper limb resistance exercises [10–12]. Additionally, it is more feasible than inter-set foot cooling in a real exercise scenario setting. Accordingly, this present study aims to investigate the effect of IPC alone and that of IPC combined with inter-set PC on increasing the number of repetitions and work volume during high-intensity resistance exercise. These measures were chosen due to their strong relevance to sports performance, as they are key indicators of an athlete’s capacity to sustain effort under heavy loads. Furthermore, as secondary outcomes, this study assessed fatigue indicators (i.e., RPE and BLA), arousal levels using the Felt Arousal Scale (FAS), and assessed NE levels during exercise. We hypothesized that IPC application could augment both the number of repetitions to exhaustion and the total work volume during high-intensity resistance exercise, potentially with additive benefits when combined with inset-set PC.

## MATERIALS AND METHODS

### Participants

Twelve resistance-trained men (mean ± standard deviation [SD]; age: 24.9 ± 4.9 years; height: 175.2 ± 6.2 cm; weight: 75.5 ± 14.3 kg, and resistance training experience 2.8 ± 1.3 years) participated in this study. The inclusion criteria required the participants to be at least 20 years old, to have engaged in resistance training at least three times per week for at least 6 months, and to be in good health with no significant medical history. The exclusion criteria were recent injury, discomfort during cooling, average blood pressure greater than 140/100 mmHg [20, 21], and the usage of ergogenic supplements or medications affecting exercise performance. The participants were instructed to maintain their usual diet but to abstain from strenuous exercise, caffeine, and alcohol for the 24 hours prior to the commencement of each workout. Additionally, they were also advised to avoid consuming a pre-workout meal 1 to 3 hours before starting each workout [11] and to skip their regular exercise routine throughout the study period.

The sample size was determined using G*Power 3.0, with an α of 0.05, a power of 0.80, and a correlation coefficient of 0.50. A previous study indicated an effect size of approximately 1 for the IPC intervention with various training volumes [22], and thus a minimum sample size of 12 was required. All the study participants were fully briefed on the experimental process, including any potential risks and benefits, and provided their informed consent before participating. This study was conducted in accordance with the principles of the Declaration of Helsinki and was approved by a local Human Research Ethics Committee.

### Experimental design

**FIG. 1:**
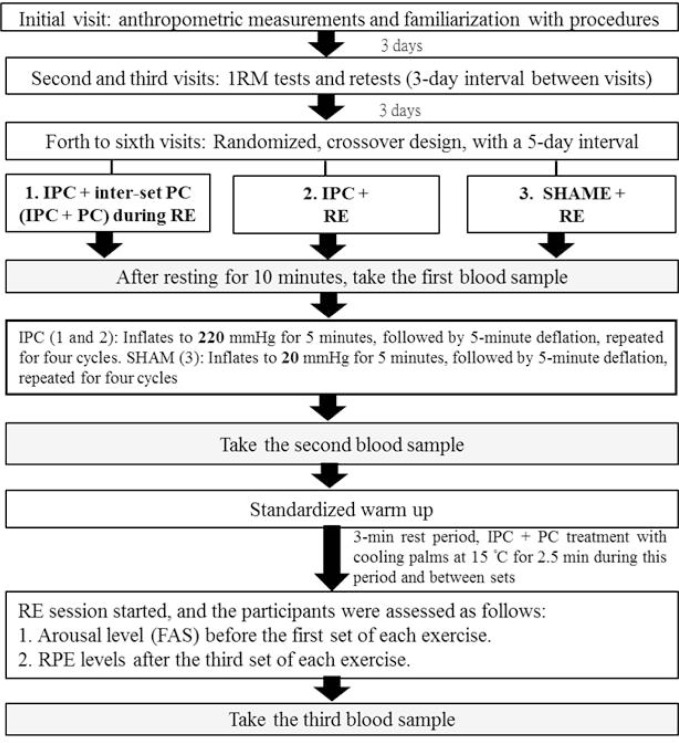
Experimental design and procedures. Note: RM = repetition maximum; IPC = ischemic preconditioning; PC = palm cooling; RE = resistance exercise; SHAM = IPC placebo control; FAS = Felt Arousal Scale; RPE = rating of perceived exertion.

This study’s experimental design is depicted in Figure 1. The initial visit involved anthropometric measurements, familiarization with procedures, and the obtainment of informed consent. Based on protocols from previous studies [8, 22], subsequent visits included conducting 1RM tests and retests for barbell BP, LP, lat pulldown (LPD), hex bar deadlift (HBD), barbell shoulder press (SP), and Smith machine back squat (SBS) exercises in this specific order, with a 3-day interval between visits.

A randomized, counterbalanced, within-group design was implemented after the 3-day interval. The participants underwent three treatments: (1) IPC + resistance exercise with inter-set PC (IPC + PC), (2) IPC + resistance exercise (IPC), and (3) SHAM (a placebo control experiment that involved applying mild pressure by wrapping a cuff) + resistance exercise.

Each of the three treatments was followed by a 5-day interval. In addition, every participant received treatment at a fixed time, and environmental conditions at the experimental site, including temperature (24–26°C) and humidity (38%–40%), were meticulously controlled throughout this study.

### Study procedures

Upon arrival at the laboratory, each participant rested for 10 minutes before a blood sample was drawn from their arm while they remained at rest. They then received either the IPC or SHAM treatment for 40 minutes under specific experimental conditions, followed by a second blood draw. Subsequently, the standardized warm-up routine described by da Silva Novaes et al. [8] was implemented, comprising two sets of 15 BP repetitions performed at 50% of the participant’s 1RM, separated by one minute of rest. The warm-up routine was followed by a 3-minute rest period before six resistance exercises were conducted in a predetermined order: barbell BP, LP, LPD, HBD, barbell SP, and SBS; each exercise was performed in three sets. Each set was performed to exhaustion at 85% of the 1RM at the maximum velocity [7], with 3-minute rest intervals between sets and exercises. Briefly, the BP was performed supine on a flat bench, lowering the barbell to the chest and pushing to full elbow extension. The LP was performed while seated, extending the knees from 90° flexion to near full extension. The LPD entailed pulling the bar to the upper chest with a pronated grip and then returning it in a controlled manner. The HBD involved standing inside the hexagonal bar and lifting it by extending the hips and knees. The shoulder press SP was performed while standing, pressing the weight overhead from shoulder level to full arm extension. Lastly, the SBS was executed using a guided bar path, squatting to at thigh-parallel depth, and then returning to full extension again. Each exercise involved multiple repetitions performed until failure, with joints to near full range of motion without locking. Success was defined as completing a repetition with a full range of motion, correct posture, and proper technique, while failure was the inability to lift the weight, improper form, or deviation from the prescribed movement pattern. For each exercise, the number of successful repetitions until failure was recorded and monitored by two experienced supervisors to ensure safety and correct execution. In IPC + PC condition, each participant immersed both their hands in 15°C ± 1°C water for 2.5 minutes before and during rest periods while performing six resistance exercises. They then rated their arousal level before each exercise utilizing the FAS [23], as well as their RPE after the third set of each exercise using the Borg CR-10 scale. A blood sample was collected at the end of the session (Figure 1).

### 1RM testing

The testing procedures and resistance exercises were modified based on previous studies [8, 22]. The warm-up and loading protocol during testing adhered to the guidelines established by da Silva Novaes et al. [8]. Briefly, each participant completed five to ten repetitions at a weight approximating 40%–60% of their 1RM, with a 1-minute rest interval between sets, for a total of two sets. The third set included three to five repetitions at 60%–80% of 1RM, followed by five maximal strength attempts to determine the actual 1RM. The inter-set rest period was 3–5 minutes, and the rest period between exercises was 10 minutes. The 1RM was determined by the maximum weight lifted with proper technique, following the same criteria as the first repetition of the resistance exercises. This approach ensured optimal posture, a full range of motion, and adherence to proper technique. During each attempt, participants were instructed to lift the weight as rapidly as possible. The maximum values from two testing sessions were utilized to set subsequent training intensities [8, 22].

### IPC and SHAM treatments

The IPC protocol in this study followed previous research regarding IPC-enhanced resistance exercise performance [8, 22], which commonly involved applying occlusion to the upper limbs regardless of whether the target exercises engaged the upper or lower limbs. A pneumatic pump inflated cuffs (57 cm long, 9 cm wide) around the upper arm near the axilla. The IPC session included four 5-minute cycles of 220 mmHg occlusion, alternating with 5 minutes of reperfusion at 0 mmHg for a total of 40 minutes. The phases alternated between arms, and participants in the SHAM condition wore cuffs pressurized to 20 mm Hg to simulate occlusion without restricting blood flow, serving as a placebo to account for psychological effects [20, 21].

### Inter-set PC intervention

The study employed the cold water immersion method [9, 13], maintaining the water level below the wrist [24]. Participants immersed their palms in 15°C ± 1°C water for 2.5 minutes, as they exhibited high tolerance to this temperature during the familiarization phase. The researchers stirred the water and added crushed ice as necessary to maintain a constant temperature [9, 13]. Participants receiving PC had their palms cooled before and during resistance exercises, with palms dried following each immersion for subsequent procedures.

### Repetitions and work volume

The numbers of repetitions of resistance exercises performed by each set of participants was documented. The work volume was determined by multiplying the total number of repetitions for each exercise by the corresponding weight. The overall work volume was calculated by adding together the training volumes for the six exercises.

### Blood sample collection and analysis

Venous blood samples were obtained three times from the nondominant forearm of each participant before and after four cycles of ischemia/reperfusion and after resistance exercise in each treatment. The blood samples were analyzed for BLa and NE levels at a clinical laboratory. The BLa levels were determined using an automated analyzer (Siemens Healthcare Diagnostics, Deerfield, IL, USA), and NE levels were analyzed using an enzyme-linked immunosorbent assay with a commercially available kit and in accordance with the manufacturer’s instructions (LDN Labor Diagnostika Nord GmbH & Co. KG, Nordhorn, Germany). The inter-assay coefficients of variation ranged from 9.2% to 10.9%, and the intra-assay coefficients of variation ranged from 11.1% to 12.8%.

### Statistical analysis

All data are presented as the mean ± SD. The intraclass correlation coefficient (ICC) indicated the test-retest reliability of 1RM maximal strength for six exercises. A one-way repeated-measures analysis of variance (ANOVA) was used to compare the total work volume and total number of repetitions between the three treatments. Significant differences were further analyzed using least significant difference (LSD) post hoc tests. A two-way repeated-measures ANOVA examined differences in blood biochemical parameters, number of repetitions, the RPE, and arousal levels during the six exercises under the various treatments. Significant condition × exercise or time interactions were subjected to detailed examination through simple main-effects tests. In instances where no significant interactions were observed, LSD post hoc tests were used to investigate the significant main effects for each condition and set. Statistical significance was set at p < 0.05. All analyses were conducted using SPSS software.

## RESULTS

A high ICC was observed for the 1RM test and retest results for all the exercises: BP (0.955), LP (0.965), LPD (0.981), HM (0.952), SP (0.943), and SS (0.961).

Significant differences in the total numbers of repetitions and total work volumes were observed in resistance exercise across different conditions (p = 0.001, effect size [ES] = 0.476 and p = 0.003, ES = 0.413, respectively). Post-hoc comparisons indicated that IPC + PC and IPC had significantly higher numbers of repetitions than did SHAM (∆ = 10.8% and 4.3% respectively), with IPC + PC surpassing IPC (∆ = 6.2%). Similarly, IPC + PC and IPC had significantly higher work volumes than SHAM (∆ = 10.7% and 2.6% respectively), with IPC + PC also exceeding IPC (∆ = 7.9%). Table 1 provides detailed numerical results and statistical comparisons, including p-values and effect sizes.

**TABLE 1:** Total training volume across 6 resistance exercises under various conditions

	Condition (each exercise includes 3 sets at 85% 1RM to exhaustion)			ANOVA (p-values/ES)	Post hoc ranking

	IPC + PC	IPC	SHAM		
Total number of repetitions	129.3 ± 21.7	121.7 ± 17.1	116.7 ± 15.9	0.001 / 0.476	IPC +PC > IPC > SHAM
Total work volumes (Kg)	11126.0 ± 3007.2	10314.10 ± 2278.5	10049.5 ± 2254.7	0.003 / 0.413	IPC +PC > IPC > SHAM

Note: RM = repetition maximum; IPC = ischemic preconditioning (220 mm Hg); PC = palm cooling; SHAM = IPC placebo control (20 mmHg).

**FIG. 2:**
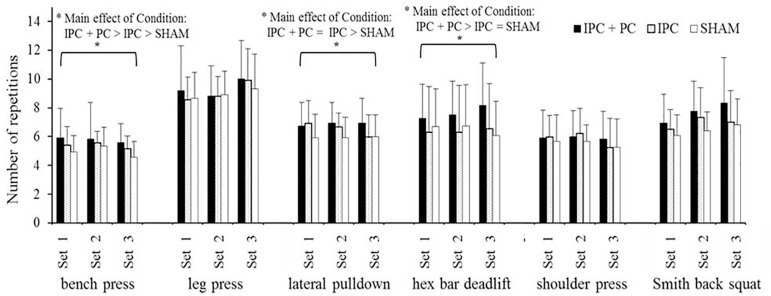
Numbers of repetitions in six resistance exercises under the IPC + PC, IPC, and SHAM conditions. IPC = ischemic preconditioning; PC = palm cooling; SHAM = IPC placebo control. * Main effect of Condition (p < 0.05).

The numbers of repetitions for BP, LPD, and HBD, shown in Figure 2, had significant main effects of Condition (p = 0.001, ES = 0.484; p = 0.025, ES = 0.286; p = 0.027, ES = 0.280). Post hoc analyses revealed he following significant differences, BP: IPC + PC > IPC > SHAM; LPD: IPC + PC = IPC > SHAM; HBD: IPC + PC > IPC = SHAM. Moreover, the numbers of repetitions for LP, SP, and SBS had no significant main effects of Condition (p > 0.05) and Exercise (p > 0.05) or a significant Condition × Exercise interaction (p > 0.05).

**FIG. 3:**
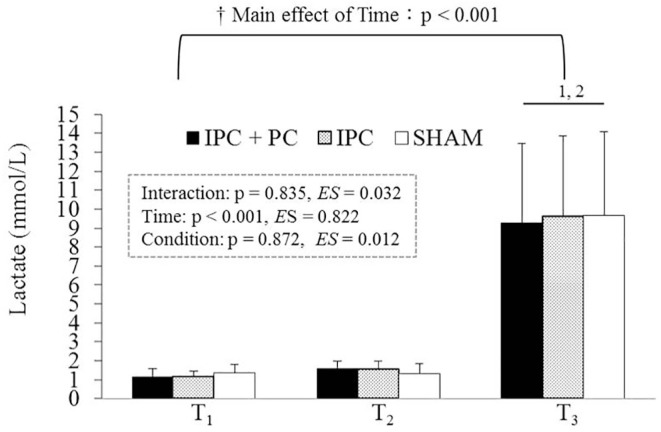
Blood lactate levels under the IPC + PC, IPC, and SHAM conditions. Note: IPC = ischemic preconditioning; PC = palm cooling; SHAM = IPC placebo control. T_1_ = before pretreatment; T_2_ = after pretreatment; T_3_ = after resistance exercise. † Main effect of Time (p < 0.05). ^1^ Significant difference with T1 (p < 0.05). ^2^ Significant difference with T2 (p < 0.05).

BLa levels exhibited a significant main effect of Time. Post hoc analysis indicated that T_3_ (post-resistance exercise) had higher levels than T_2_ (post-pretreatment) and T_1_ (before pretreatment) under all conditions (Figure 3).

**FIG. 4:**
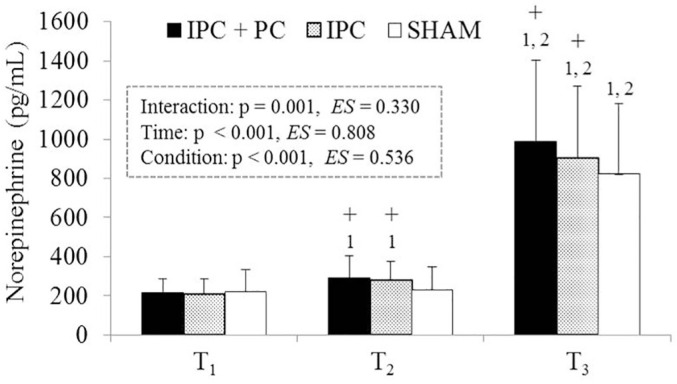
Blood norepinephrine levels under the IPC + PC, IPC and SHAM conditions. Note: IPC = ischemic preconditioning; PC = palm cooling; SHAM = IPC placebo control. T_1_ = before pretreatment; T_2_ = after pretreatment; T_3_ = after resistance exercise. † Main effect of Time (p < 0.05). + Significant difference with SHAM (p < 0.05). ^1^ Significant difference with T_1_ (p < 0.05). ^2^ Significant difference with T_2_ (p < 0.05).

As shown in Figure 4, no significant differences in NE levels among IPC + PC, IPC, and SHAM at T_1_ (p = 0.293, ES = 0.106). NE levels were significantly higher for IPC + PC and IPC than for SHAM at T_2_ (p = 0.001 and p = 0.003, respectively) and T_3_ (p = 0.003 and p = 0.021, respectively). In addition, NE levels were significantly higher at T_3_ than at T_2_ (p < 0.001) and T_1_ (p = 0.003) across all the conditions. However, a significant increase was observed at T_2_ compared with T_1_ (p = 0.005) only under the IPC + PC (p = 0.014) and IPC conditions (p = 0.015).

As for perceptual parameters, RPE value had no significant main effects of Condition (p > 0.05) and Exercise (p > 0.05) as well as the Condition × Exercise interaction (p > 0.05) (Table 2). However, FAS score had significant main effects of Condition (post hoc comparisons: IPC + PC > IPC > SHAM, Table 3) and Exercise.

**TABLE 2:** Rating of perceived exertion after each exercise under various conditions

condition	6 exercises, consisting of 3 sets at 85% 1RM to exhaustion						ANOVA (p-value/ES)		

	BP	LP	LPD	HBD	SP	SBS	interaction	condition	exercise
IPC + PC	8.3 ± 0.6	8.3 ± 0.7	8.0 ± 0.7	7.9 ± 0.5	8.2 ± 0.6	8.1 ± 0.7	0.261/0.103	0.232/0.124	0.172/0.128
IPC	8.3 ± 0.5	8.1 ± 0.5	8.4 ± 0.5	7.9 ± 0.7	7.9 ± 0.8	8.2 ± 0.4			
SHAM	8.0 ± 0.3	7.8 ± 0.7	8.1 ± 0.5	7.8 ± 0.6	8.1 ± 0.3	8.3 ± 0.5

Note: RM = repetition maximum; IPC = ischemic preconditioning (220 mm Hg); PC = palm cooling; SHAM = IPC placebo control (20 mm Hg); BP = bench press; LP = leg press; LPD = lat pulldown; HBD = hex bar deadlift; SP = shoulder press, SBS = Smith machine back squat.

**TABLE 3:** Felt Arousal Scale scores before each exercise under various conditions

condition	6 exercises, consisting of 3 sets at 85% 1RM to exhaustion						ANOVA (p-value/ES)		

	BP	LP	LPD	HBD	SP	SBS	interaction	condition	exercise
IPC + PC	3.2 ± 0.4	3.4 ± 0.5	3.2 ± 0.4	3.8 ± 0.7	3.7 ± 0.5	3.6 ± 0.7	0.912/0.040	0.001/0.463	< 0.001/0.364
IPC	2.8 ± 0.4	3.3 ± 0.5	3.2 ± 0.4	3.7 ± 0.7	3.4 ± 0.7	3.3 ± 0.5			
SHAM	2.9 ± 0.5	3.2 ± 0.4	3.0 ± 0.4	3.4 ± 0.7	3.1 ± 0.3	3.3 ± 0.5
Condition effect post hoc ranking (p values and ES): IPC + PC > IPC > SHAM (p = 0.01; ES = 0.5)									

Note: RM = repetition maximum; IPC = ischemic preconditioning (220 mm Hg); PC = palm cooling; SHAM = IPC placebo control (20 mm Hg); BP = bench press; LP = leg press; LPD = lat pulldown; HBD = hex bar deadlift; SP = shoulder press, SBS = Smith machine back squat.

## DISCUSSION

The primary findings revealed that IPC increased the number of repetitions by 4.3% and augmented the work volume by 2.6% over a single high-intensity resistance exercise session. Furthermore, combining inter-set PC with IPC resulted in a 10.8% increase in the number of repetitions and a 10.7% increase in the work volume. IPC increased the number of repetitions in BP and LPD exercises, with additional benefits in BP, LPD, and HBD exercises when interset PC was added. Moreover, administering inter-set PC following IPC had an ergogenic effect on the numbers of repetitions in the BP and HBD exercises compared with the application of IPC alone. These findings suggest that both IPC and IPC combined with PC may serve as practical, time-efficient, and non-invasive strategies for athletes and coaches aiming to increase training volume acutely and optimize performance outcomes during high-intensity resistance sessions.

This study extends those of da Silva Novaes et al. [8] by employing a higher-intensity protocol (85% 1RM instead of 80% 1RM). The results demonstrated that IPC on the upper arm can boost performance in resistance exercise work volume; this finding was in line with those of previous studies [8, 22]. Our findings, however, were marginally different from those of da Silva Novaes et al., who noted an ergogenic effect in five out of six consecutive resistance exercises after IPC, resulting in a 26% increase in overall work volume. In our study, a greater number of repetitions were noted in most of the resistance exercises post-IPC application compared with post-SHAM. However, an inter-individual difference was observed; some participants experienced an initial increase in the number of repetitions but struggled to adequately recover for subsequent sets, resulting in fewer repetitions than those who received SHAM treatment. Consequently, significant differences were observed only in the initial phase exercises of the upper body, namely BP and LPD, and the total work volume increased by 2.6% when compared to those who received SHAM treatment. This finding was comparable to that of a recent study indicating that IPC is effective in enhancing repeated power performance during the initial phase, with local IPC exhibiting superior effectiveness to remote IPC [25]. Additionally, the relatively minor gains in exercise benefits observed in the present study compared with da Silva Novaes et al. [8] could have been owing to the higher intensity and longer rest intervals between sets in our study. The potential ergogenic effects of IPC on repeated resistance exercise include augmentation of reactive hyperemia blood flow [5], muscle perfusion and oxygen extraction capacity [6], and the buffering of acidosis [7]. These effects appear to be particularly favorable for low-intensity resistance exercises performed to exhaustion with brief rest periods between sets, considering the metabolic demands and oxygen availability that are typically encountered during such exercises challenges [26, 27]. In the present study, we set an intensity level at 85% of 1RM, with specific emphasis on improving neural excitability during states of fatigue rather than enhancing oxygen availability alone [28]. These findings imply that the benefits of IPC on oxygen extraction and buffering capacities may be restricted during high-intensity resistance exercise with longer rest intervals, rendering it less effective than exercising at 80% of 1RM. Nonetheless, it still demonstrates a modest ergogenic effect.

The present study detected no significant rise in BLa levels after four IPC cycles. There was no discernible difference in blood BLa levels between the participants who received IPC treatment and those who received SHAM treatment, which was consistent with findings described by Bailey et al. [29]. This suggests that no muscle fatigue occurred before resistance exercise in the present study, since BLa is commonly employed as a measure of peripheral fatigue [7]. Unexpectedly, after four cycles of IPC, a minor but significant increase in NE was noted. The ischemic method of IPC is analogous to the 15-minute blood flow restriction on non-exercised limbs, which was also reported to induce a significant rise in NE [30]. NE is a neurotransmitter released primarily from sympathetic nerves, acting as an index of sympathetic nervous system drive and arousal [19]. Increased NE levels observed after IPC reinforce the notion that IPC modifies sympathetic activation [31]. Aroused brain regions play a role in motor command and force production, which might boost performance in sports requiring maximal power, potentially improving performance in stimulating situations [32]. Higher arousal levels have been linked to greater number of repetitions during high-intensity resistance exercises [33]. In the present study, IPC elevated NE levels before and after resistance exercise relative to the SHAM treatment (Figure 4), along with higher arousal levels throughout the exercise (Table 2). Collectively, in addition to previously proposed candidate mechanisms [5–7], as indicated in the present study, an IPC-induced increase in NE levels before the resistance exercise can create favorable conditions for enhancing force production and thus could partially contribute to the ergogenic mechanism.

Performing inter-set PC can enhance performance in various resistance exercises, including BP [10], LP [11], and pull-up [12] exercises. This study is the first to explore the combined effect of IPC and inter-set PC on high-intensity resistance exercise. The findings indicated that compared with the SHAM condition, IPC + PC application led to notably higher NE before and after exercise and a higher FAS score during exercise. Additionally, an overall increase in work volume of 10.8% was observed, as was improved performance in exercises targeting major muscle groups of the upper and lower body that require a firm grip, such as BP, LPD, and HBD exercises. In addition, this synergistic effect augmented the ergogenic benefits of IPC. The observed physiological responses to IPC + PC treatment were mostly more significant than those to IPC alone, contributing to an additional increase of 7.9% in total work volume and repetitions in BP and HBD exercises. Although the exact mechanisms remain to be fully elucidated, one plausible explanation involves arousal-mediated enhancement of NE elevation, which may boost the efficiency of motor unit recruitment [19] and prove beneficial in tasks requiring maximal effort [32]. Prior research has established that elevating arousal levels [9, 13] and NE [14] through peripheral cooling can benefit strength performance. The combined application of IPC and PC in the present study further augmented arousal levels, as indicated by the increased NE levels prior to exercise and increased FAS scores during exercise. These findings imply that the enhanced arousal levels elicited by the combined application of IPC and interset PC may represent a partial mechanism underlying the observed enhancements in resistance exercise performance.

Individuals engaging in high-volume resistance training often experience considerable fatigue due to the demanding workload [34]. The present study found the work volume ranking to be IPC + PC > IPC > SHAM, with no significant difference observed in fatigue markers among the interventions. Carvalho et al. [**7**] obtained similar results, indicating that IPC application did not impact fatigue markers more significantly than did SHAM condition. Previous studies have indicated that increasing arousal levels not only boosts force production but also reduces the effort sensation, facilitating performance of more intense exercise without causing discomfort [32]. The present study suggests that IPC application, alone or in combination with PC, may improve performance and potentially mitigate fatigue during resistance exercise, as evidenced by elevated arousal levels without a significant rise in fatigue indicators, despite a higher work volume being completed.

This study has certain limitations that need to be acknowledged. This study highlights the benefits of single resistance exercise, but reservations remain about the rapid adaptation to ischemic and cooling tolerance, the potential requirement for additional stimuli, and the preservation of cumulative training effects. Further research is needed to understand the long-term effects of implementing IPC, either alone or in conjunction with inter-set PC during resistance training.

## CONCLUSIONS

Applying upper-arm IPC can augment the work volume in a single high-intensity resistance exercise session, particularly during the early phases of upper body exercises. Furthermore, incorporating inter-set PC into IPC enhances most of the ergogenic effects of IPC, particularly for exercises that require a firm grip, such as BP and HMD. The enhancement of high-intensity resistance exercise performance may be partially attributed to the amplified arousal response resulting from inter-set PC and the heightened arousal level caused by IPC before and during the resistance exercise. Finally, applying IPC alone or that with inter-set PC may help mitigate the sensation of fatigue during resistance exercise. In conclusion, this finding, along with previous research demonstrating that IPC can promote recovery, mitigate muscle damage, and regulate inflammatory and oxidative stress responses associated with strength training [35], offers a basis for further investigation into the short-term effects of training, with a focus on muscle functional performance.
